# Biochemical Characterization of an In-House *Coccidioides* Antigen: Perspectives for the Immunodiagnosis of Coccidioidomycosis

**DOI:** 10.3390/molecules17077854

**Published:** 2012-06-28

**Authors:** Renato Evando Moreira Filho, Silviane Praciano Bandeira, Raimunda Sâmia Nogueira Brillhante, Marcos Fábio Gadelha Rocha, Ilka Maria Vasconcelos, Mirella Leite Pereira, Débora de Souza Collares Maia Castelo-Branco, Francisco Airton Castro Rocha, Zoilo Pires de Camargo, Marcio Viana Ramos, Rossana de Aguiar Cordeiro, José Júlio Costa Sidrim

**Affiliations:** 1 Specialized Medical Mycology Center, School of Medicine, Federal University of Ceará, Fortaleza, CE, CEP, 60430-270, Brazil; 2 Postgraduate Program in Veterinary Sciences, School of Veterinary Medicine, Ceará State University, Fortaleza, CE, CEP, 60740-000, Brazil; 3 Department of Biochemistry and Molecular Biology, Federal University of Ceará, Pici Campus, Fortaleza, CE, 60455-760, Brazil; 4 Department of Microbiology, Immunology and Parasitology, São Paulo Federal University, São Paulo, 04021-001, Brazil

**Keywords:** coccidioidomycosis, immunodiagnosis, antigens, β-glucosidase, glutamine

## Abstract

The objective of this study was to evaluate the reactivity of an in-house antigen, extracted from a strain of *C. posadasii* isolated in northeastern Brazil, by radial immunodiffusion and Western blotting, as well as to establish its biochemical characterization. The protein antigen was initially extracted with the use of solid ammonium sulfate and characterized by 1-D electrophoresis. Subsequently, it was tested by means of double radial immunodiffusion and Western blotting. A positive reaction was observed against the antigen by both immunodiagnostic techniques tested on sera from patients suffering from coccidioidomycosis. Besides this, two immunoreactive protein bands were observed and were revealed to be a β-glucosidase and a glutamine synthetase after sequencing of the respective N-terminal regions. Our in-house *Coccidioides* antigen can be promising as a quick and low-cost diagnostic tool without the risk of direct manipulation of the microorganism.

## 1. Introduction

Coccidioidomycosis is a predominantly pulmonary disease, with systemic manifestations, caused by the dimorphic fungi *Coccidioides immitis* and *C. posadasii*, acquired after inhalation of their infective conidia [1]. Severe manifestations of the disease are generally observed in immunocompromised patients, as well as patients suffering from chronic degenerative diseases, such as type 2 diabetes mellitus [2]. Clinical diagnosis of the disease can be difficult and cultivation of the microorganism is slow and risky, since direct manipulation of the fungal arthroconidia requires special care in the laboratory [3,4]. Recent studies have focused on searching for faster and safer diagnostic methods, such as immunodiagnosis and molecular biology techniques [5].

Detection of antigens can be useful for early and safe diagnosis of the pathogen, since antigenemia has been observed in 56–78% of patients, in recent studies [3]. Therefore, serological tests have been used to avoid the risky manipulation of the microorganism and to reduce the time and cost of testing [6].

Previous studies have demonstrated that despite genotype differences among *Coccidioides* spp, it is possible to obtain antigen extracts for the performance of serological tests from a single strain of the fungus [7]. For example, Brilhante *et al*. [8] described the potential use of an antigen produced from a single *Coccidioides posadasii* strain for serological diagnosis of coccidioidomycosis. Besides this, these authors showed it is possible to employ an in-house antigen when a false negative result is obtained from the use of a widely used commercial antigen.

In this context, the aim of this study was to assess the reactivity of an in-house antigen extracted from a *C. posadasii* strain isolated in northeastern Brazil by radial immunodiffusion and Western blotting, as well as to establish the antigen’s biochemical characterization. 

## 2. Results and Discussion

The double immunodiffusion verified that the antigen, at a concentration of 54.5 µg/100 µL, showed positive reactions to all the serum samples from patients with coccidioidomycosis. The serum used as positive control (Immy Immunodiagnostics) also reacted positively with the antigen tested. There was no positive reaction with the serum samples from patients with paracoccidioidomycosis, histoplasmosis and aspergillosis.

Concerning the reactivity of the sera from patients suffering from deep mycosis to the antigen tested using the Western blot technique, there were positive results with all the serum samples from patients with coccidioidomycosis, with reagent bands between 35 and 100 kDa. No reaction was observed with sera from patients with paracoccidioidomycosis or aspergillosis, but there was a cross-reaction with 3 serum samples from patients with histoplasmosis, as well-known in coccidioidomycosis immunodiagnosis, even when purified antigens are used [9].

**Figure 1:**
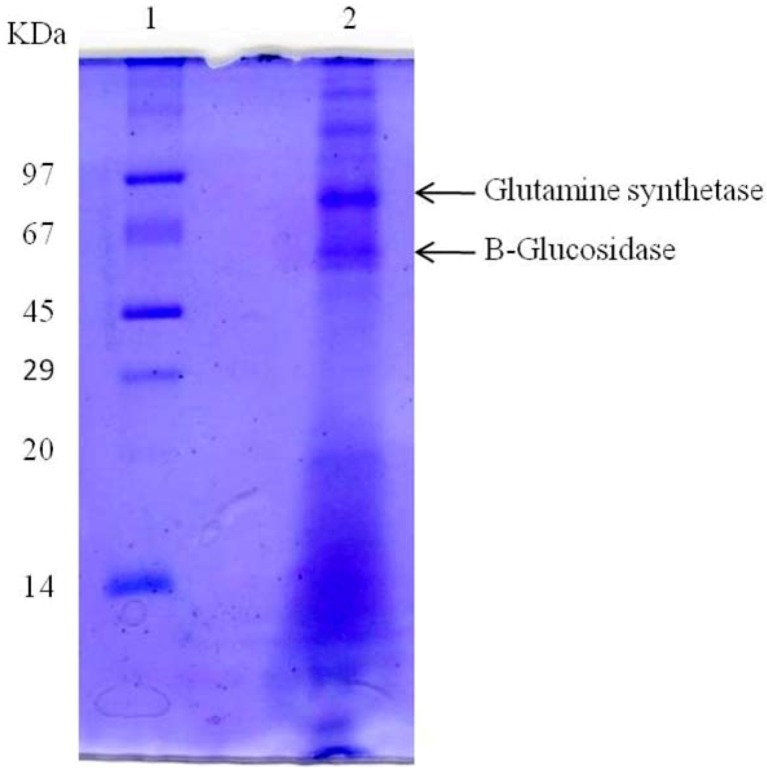
SDS-PAGE—In house antigen (34 μg) were loaded on a 15% polyacrylamide gel and resolved with a constant current of 20 mA for approximately 2 h. Protein bands were stained with Coomassie Blue R250. Lane 1, molecular mass markers (Phosphorylase b—97 kDa; bovine serum albumin—66 kDa; ovalbumin—45 kDa; carbonic anhydrase—29 kDa; soybean trypsin inhibitor—20.1 kDa and lactalbumin—14.4 kDa); Lane 2: in-house antigen.

Denaturating electrophoresis of the in-house antigen showed the presence of several protein bands. Among the protein bands detected, two bands located in the ranges of 45–67 kDa and 67–97 kDa were well defined, having been designated 45–67 kDa band and 67–97 kDa band, respectively (Figure 1). In turn, the protein bands observed by the same technique when analyzing the antigen produced by Immy Immunodiagnostics showed a different molecular weight, situated between 15 and 35 KDa.

The N-terminal amino-acid sequence of the 45–67 kDa band was AGKLGFALGVKNADGSCKS, whereas the 67–97 kDa band was NAHYVLAAIVALGWRG. Search for homology in the primary structure showed 100% similarity of the 45–67 kDa band and 67–97 kDa band with a β-glucosidase and a glutamine synthetase, respectively, of *Coccidioides posadasii* (Table 1).

**Table 1:** Comparison of N-terminal sequences of 45–67 kDa and 67–97 kDa bands of in house antigen with similar protein sequences.

Protein	Species	Amino acid sequence	% Similarity	Accession number
45–67 kDa band (this report)	*Coccidioides posadasii*	^1^AGKLGFALGVKNADGSCKS^19^		
β-Glucosidase *	*Coccidioides posadasii*	^20^AGKLGFALGVKNADGSCKS^38^	100	XP_003070157
67–97 kDa band (this report)	*Coccidioides posadasii*	^1^NAHYVLAAIVALGWRG^16^		
Glutamine synthetase *	*Coccidioides posadasii*	^403^NAHYVLAAIVALGWRG^418^	100	XP_003072037

* NCBI Blast (www.ncbi.nlm.nih.gov).

Several experiments have been performed to develop potentially useful serological assays for the immunodiagnosis of coccidioidomycosis [10]. The use of antigens from local strains is important to improve diagnostic accuracy because, as described by Brilhante *et al*. [8], occasionally, the commercially available antigen is not able to detect the presence of anti-*Coccidioides* antibodies. However, antigens extracted from Brazilian strains have not been widely used yet, probably because of the absence of large-scale production and standardization of the use of these specific antigens. Brilhante *et al*. [8] suggested that the association of cultivation techniques, PCR reactions and serological tests can replace conversion in animals to confirm infection by *Coccidioides* spp. Moreover, the utilization of various serological techniques increases the sensitivity of laboratory diagnosis, even among immunocompromised patients [11].

Protein precipitation with ammonium sulfate is known for being useful to concentrate diluted protein solutions and to fractionate protein mixtures. For the present study we selected the method developed by the Specialized Medical Mycology Center (CEMM) of Ceará Federal University, Brazil [8]. This is a simple, easily performed and low-cost technique. Indeed, various antigen fractions have been used in serological tests to diagnose coccidioidomycosis, with varied protocols for extracting antigens from *C. immitis* [12,13,14,15].

Our research group has been studying the immunological diagnosis of coccidioidomycosis through immunodiffusion, ELISA and Western Blot using our in-house antigen obtained from a local strain of *C. posadasii*, resulting in an approximate value for sensitivity and specificity of 85% and 95%, respectively (data not shown). The data concerning the results obtained through ELISA are not presented in this paper because they are part of a broader project that is in the final stage of conclusion. In the present work, this in-house *Coccidioides* antigen was used in immunodiffusion assays against serum samples from patients suffering from coccidioidomycosis, aspergillosis, paracoccidioidomycosis and histoplasmosis along with a commercial serum positive for coccidioidomycosis produced by Immy Immunodiagnostics. The in-house antigen reacted with all the serum samples from patients with coccidioidomycosis but not with those from patients with the other mycoses. These results are in accordance with the estimated sensitivity and specificity for this method, when applying the in-house antigen. In addition, there are prospects to use immunodiffusion in epidemiological inquiries in endemic and non-endemic areas, by using antigens from strains that are local to the region under investigation. 

Western blotting is often used to confirm whether experimental protein antigens can be used to diagnose patients suffering from other diseases [16]. The reaction with our antigen using this technique was positive for all sera from coccidioidomycosis patients, which demonstrates that the in-house antigen affords a high sensitivity of this immunological assay. Regarding the sera from patients with other fungal infections, samples from histoplasmosis patients cross-reacted with the in-house antigen, possibly, because Western blotting is more sensitive than double radial immunodiffusion. In turn, the serum samples from patients infected with paracoccidioidomycosis and aspergillosis were negative. The occurrence of cross-reactions when using highly sensitive immunoenzymatic techniques like Western blotting is documented in the literature [17,18]. This drawback can result from impurities in the antigen sample, the ample sharing of cell wall components between these fungi and the possibility of previous contact of the individual with people carrying more than one of the analyzed pathogens [19]. These findings suggest that immunodiffusion can be useful for initial immunodiagnosis of patients with clinical symptoms, under epidemiological situations that suggest the occurrence of coccidioidomycosis. Even though this technique presents higher specifity than sensibility because of its limitation to detect immunocompromised patients and those with very early primary infection, it is preferred as a first-line diagnostic method since it is highly feasible and does not require special equipments. On the other hand, skin-based coccidioidin tests are known to be more sensitive since they can detect patients with early primary infection [20]. However, it was not possible to perform a skin test with coccidioidin on the selected patients, since this antigen is not available in Brazil [18]. Additionally, detection of fungal antigens has been reported for the diagnosis of coccidioidomycosis. More recently, a specific *Coccidioides* antigen ELISA was developed, with a sensitivity of 71% and specificity of 98%, in patients who had underlying immunosuppression and moderate to severe coccidioidomycosis. However, cross-reactivity occurred in approximately 10% of patients who had other endemic mycoses, such as histoplasmosis [9].

In the next step of the study, we performed biochemical analysis of the protein antigen tested. We first evaluated the electrophoretic profile by SDS-PAGE and staining with Coomassie brilliant blue R-250. There were two protein bands produced, compatible with the immunoreactive zone observed in the Western blotting tests, with molecular weight ranging from 45 to 97 KDa. This finding agrees with those of previous studies, which have described the detection of protein antigens of the *Coccidioides* genus, most presenting molecular weights varying between 20 and 100 KDa [10,21,22,23].

Once the electrophoretic profile of the antigen tested was identified, two protein bands were transferred to determine if their N-terminal sequencing were related to already known proteins. The analysis of the amino acids from the protein band with molecular weight between 45–67 KDa (AGKLGFALGVKNADGSCKS) as well as from the protein band between 67–97 KDa (NAHYVLAAIVALGMEG) revealed homology, respectively, with a β-glucosidase and a glutamine synthetase.

β-glucosidases are exocelulases that cleave disaccharide bonds or glucose-substituted molecules [24]. They are involved in the metabolic activity (e.g., hydrolysis of cellulose and polysaccharides) of various organisms, such as bacteria [25]; insects [26]; plants [27] and fungi [28]. Their participation in the metabolism of *Coccidioides immitis* is relevant because they are essential to cleave the cell wall of the fungal spherule, contributing to the plasticity and growth of this form of the fungus. The use of β-glucosidase in serological techniques to diagnose coccidioidomycosis has been described, such as the precipitin tube test [29], suggesting its use in the immunodiagnosis of coccidioidomycosis. It should be borne in mind that in patients infected with *Coccidoides* sp., this microorganism divides continually. The development of a technique for easy extraction of a β-glucosidase, with application in the immunodiagnosis of coccidioidomycosis, can be promising as a quick and low-cost diagnostic method without the risk of direct manipulation of the microorganism.

Glutamine synthetases, in turn, are proteins responsible for catalyzing the reaction between ammonia and glutamic acid, generating glutamine [30]. They are the main metabolic route for ammonia fixation by fungi during tissue growth, in the presence of nitrate [31]. This biochemical process is critical for the majority of geophilic microorganisms. In fungi, glutamine is an essential amino acid, playing a central role in the metabolism of nitrogen. It is a precursor of various essential metabolites, such as nucleic acids and nitrogenated carbohydrates, as well as other amino acids, like histidine, tyrosine and asparagin [32]. The beneficial metabolic activity promoted by this enzyme, during division in the host, allows to infer that it is continually expressed during infection with *Coccidioides* spp. Additionally, the antigenic potential of this protein, for the diagnosis of coccidioidomycosis, has not been described yet.

## 3. Experimental

### 3.1. Selection of the Fungal Strains

Twelve strains of *C. posadasii* were analyzed, obtained from the collection of the Specialized Medical Mycology Center (CEMM) of Ceará Federal University (UFC), to determine their protein concentration by the method described by Bradford [33], utilizing bovine serum albumin (BSA). From these, strain CEMM 05-2-063 was selected, because it had a higher protein concentration than the others.

### 3.2. Obtaining and Fractionating the Protein Antigen

To obtain the antigen, through the protocol described by Brilhante *et al.* [8], with slight modification, the *C. posadasii* strain was maintained in the mycelial phase and cultivated in a medium containing 2% glucose and 1% yeast extract for 45 days, at 30 °C, without agitation. Then the culture was inactivated with 0.2 g/L of thimerosal (Synth, São Paulo, Brazil) during 20 days and the supernatant was filtered through filter paper. The proteins from the sample were precipitated by adding solid ammonium sulfate (Sigma Chemical Corporation, St. Louis, MO, USA), to obtain an antigen in a saturation interval of 0–90% (total antigen) according to Scopes’ formula [34]. The solution was maintained at 4 °C for 24 h, with subsequent recovery of the protein precipitate by centrifugation at 12,000 ×g for 30 min. Then exhaustive dialysis was performed with distilled water in a membrane with a cutoff of 10 kDa and the resulting material was stored at −20 °C until use. On being removed from storage, the antigen samples were lyophilized and submitted to a new preparation process by means of mixture with polyvinylpyrrolidone [35].

### 3.3. Evaluation of the Antigen Immunoreactivity by the Double Radial Immunodiffusion Technique (Ouchterlony)

Double radial immunodiffusion assays were performed, as described elsewhere [36]. The extracted antigen was tested against serum samples from patients with coccidioidomycosis (n = 14), histoplasmosis (n = 7), paracoccidioidomycosis (n = 5) and aspergillosis (n = 5). The definite diagnosis of these mycoses was based on fungal recovery from clinical specimens. Additionally, positive serum (with anti-*Coccidioides immitis* antibodies) and a commercial antigen produced by Immy Immunodiagnostics (Norman, OK, USA) were used as controls.

### 3.4. Evaluation of the Antigen Immunoreactivity by the Western Blot Technique

The antigen samples were separated by electrophoresis (SDS-PAGE) according to the protocol described elsewhere [37]. Then the protein contained in the electrophoretic gel was transferred to a nitrocellulose membrane in a semi-dry system, with a current of 65 mA, during 60 to 120 min. After that, the nitrocellulose membrane was cut into 4 mm × 8 mm strips, which were blocked with a buffer consisting of Tris-NaCl-skim milk-goat serum and the strips with the test serum (1:200) were incubated for 18 h under agitation. After successive washings with 0.01% Tris-NaCl-Tween 20 buffer, the strips were incubated with anti-IgG conjugate marked with alkaline phosphatase diluted to 1:500 for 90 min, with subsequent addition of the substrate nitro blue tetrazolium/bromochloroindolyl phosphate (NBT/BCIP). The presence of the antigen-antibody reaction was detected by the formation of a brownish band [38]. Test sera were used from patients suffering from coccidioidomycosis (n = 10), histoplasmosis (n = 5), paracoccidioidomycosis (n = 5) and aspergillosis (n = 5), besides serum from a healthy individual as negative control. 

### 3.5. Biochemical Analysis

#### 3.5.1. Polyacrylamide Gel Electrophoresis (SDS-PAGE)

The electrophoresis was carried out under denaturing conditions (SDS-PAGE) using the discontinuous method described elsewhere [37]. A 15% polyacrylamide gel in 0.025 M Tris-HCl, pH 8.8, containing 0.2 M glycine and 1% sodium dodecyl sulphate (SDS) was mounted in a vertical electrophoresis system. Sample and molecular mass standards were prepared in 0.5 M Tris-HCl buffer, pH 6.8, containing 0.1% SDS, and boiled at 98 °C for 10 min before loading on the gel. Electrophoresis was performed with a constant current of 20 mA for approximately 2 h. Protein bands were stained with Coomassie Blue R250. For comparison, the same technique was used to analyze the commercial antigen produced by Immy Immunodiagnostics.

#### 3.5.2. Determination of N-Terminal Sequencing

The N-terminal amino-acid sequences of 45–67 kDa and 67–97 kDa bands visualized by SDS-PAGE were determined on a Shimadzu PPSQ-23A Automated Protein Sequencer (Tokyo, Japan) performing Edman degradation. Sequences were determined from 45–67 kDa and 67–97 kDa bands blotted on polyvinylidene difluoride (PVDF) membrane after SDS-PAGE. PTH amino-acids were detected at 269 nm after separation on a reversed phase C_18_ column (4.6 × 2.5 mm^2^) under isocratic conditions, according to the manufacturer’s instructions [39]. Searches for sequence similarity were performed with the BLASTp program [40].

## 4. Conclusions

A positive reaction was observed against the antigen by radial immunodiffusion and Western blotting on sera from patients suffering from coccidioidomycosis and β-glucosidase and a glutamine synthetase were revealed to be the main immunoreactive proteins. Therefore, our in-house *Coccidioides* antigen can be promising as a quick and low-cost diagnostic tool without the risk of direct manipulation of the microorganism.
